# Unplanned admissions after day-case surgery in an Italian third-level pediatric hospital: a retrospective study

**DOI:** 10.1186/s13741-023-00342-y

**Published:** 2023-09-26

**Authors:** Alessandro Vittori, Luigi Tritapepe, Fabrizio Chiusolo, Emanuele Rossetti, Marco Cascella, Emiliano Petrucci, Roberto Pedone, Franco Marinangeli, Elisa Francia, Ilaria Mascilini, Giuliano Marchetti, Sergio Giuseppe Picardo

**Affiliations:** 1 Department of Anesthesia and Critical Care, ARCO ROMA, Ospedale Pediatrico Bambino Gesù IRCCS, Piazza S. Onofrio 4, 00165 Rome, Italy; 2 Department of Anesthesiology, Critical Care, and Pain Medicine, San Camillo Forlanini Hospital, Circonvallazione Gianicolense 87, 00152 Rome, Italy; 3 Unit of Anesthesia and Intensive Care, Sapienza University, Piazzale Aldro Moro 5 00185, Rome, Italy; 4 Department of Anesthesia and Critical Care, Istituto Nazionale Tumori-IRCCS, Fondazione Pascale, Via Mariano Semmola, 53, 80131 Naples, Italy; 5 Department of Anesthesia and Intensive Care Unit, San Salvatore Academic Hospital of L’Aquila, Via Lorenzo Natali, 1, 67100 Coppito, L’Aquila Italy; 6 Department of Psychology, University of Campania Luigi Vanvitelli, Viale Abramo Lincoln, 5, 81100 Caserta, Italy; 7 Department of Anesthesiology, Intensive Care and Pain Treatment, University of L’Aquila, , L’Aquila, Piazzale Salvatore Tommasi, 1, 67100 Coppito, AQ Italy; 8 Surgery Unit, Bios Medical Center, Via Domenico Chelini, 39, 00197 Rome, Italy

**Keywords:** Day-case surgery, Pediatric anesthesia, Unplanned admission, Pain, Children, Ambulatory anesthesia

## Abstract

**Background:**

Increasing procedures in day-case surgery can mitigate the costs of health service, without reducing safety and quality standards. The Ospedale Pediatrico Bambino Gesù has adopted an educational program for healthcare personnel and patients’ families to increase the number of day-case surgery procedures performed without reducing the level of safety. The unplanned admission rate after day-case surgery can be a quality benchmark for pediatric day-case surgery, and in literature, there are no Italian data.

**Methods:**

We made a retrospective analysis of the hospital database and focused on children requiring unplanned admission to the central venue of the hospital for the night. The audit covered the period from September 2012 to April 2018.

**Results:**

We performed general anesthesia for 8826 procedures (urology 33.60%, plastic surgery 30.87%, general surgery 17.44%, dermatology 11.66%, dentistry 3.16%, orthopedics 1.64%, digestive endoscopy 1.63%). Unplanned admission for anesthetic reasons resulted in two cases: one case of syncope and one case of vomit (0.023% rate). No one major complication.

**Conclusions:**

Good quality of patient selection, the safety of the structure, family education, and an efficient organizational model combined with an educational program for anesthesiologists can improve the safety of anesthesia for day-case surgery.

## Background

Reducing the costs of health services is a priority in every country, and it is an important aim for hospitals without decreasing the quality of assistance and safety (Gandhi et al. 2018). Increasing the percentage of day-case procedures by defining eligible patients and procedures is one option to reduce the costs of hospitalization (Stabile et al. 2013; Torre and Federici 2017; Caredda et al. 2019). Aims of day-case surgery are applicable in pediatrics too, with additional advantages: decreased risk of disease transmission, reduced stress for children and their families, and downplaying parental separation (Bailey et al. 2019).

Furthermore increasing day-case surgery can maximize hospital procedures volume, which is a benchmark of the hospital’s qualitative (Fellin et al. 1995; Cua et al. 2017).

Although it is very challenging to combine safety and feasibility in low surgical procedures without exceeding in terms of costs and day-case surgery, it should reduce as much as possible major complications (bronchospasm, laryngospasm, hemodynamic instability, anaphylaxis) and minor complications as postoperative nausea and vomiting (PONV) and pain (Habre et al. 2017; Wolfler et al. 2020). In addition, logistic criteria and socio-economic reasons sometimes preclude day-case surgery setting for some patients: low understanding or the hospital rules by the family and a poor socioeconomic background of family to support the children (Luca et al. 2018). However, day-case surgery in the pediatric field must provide for a strong collaboration of families as the management of postoperative pain is managed at home.

A measure of quality and safety of day-case anesthesia can be the incidence of unplanned hospital admissions and which causes can determinate these events (Gold et al. 1989).

In 2012, Ospedale Pediatrico Bambino Gesù, a third-level pediatric hospital, started pediatric day-case surgery in a new location, called “San Paolo,” outside the historical headquarter and opened only during daytime (Bambin and Gesù: a San Paolo apre nuova sede per i più piccoli. n.d.). In this structure, there are no wards, intensive care unit, and blood establishment, while for emergency, there is a protocol to transfer patients in the main hospital. Before opening this new venue, the Ospedale Pediatrico Bambino Gesù began an educational program for healthcare personnel, especially anesthesiologists, and patients’ families to reconcile the increase in the volume of procedures and patient safety. The primary aim of this study was to investigate the incidence of unplanned admissions following pediatric day-case surgery for anesthetic reasons. In fact, there are currently no Italian pediatric data on the incidence and reasons of unplanned admission after day-case surgery. Furthermore, our study focuses on the anesthetic reasons for unplanned admission after day-case surgery and describes in detail an organizational model that can also be applied in other centers. The system level of patient safety is a remarkable topic in pediatric anesthesia and requires research and training of anesthesiologists These two original data fill a literature gap regarding the topic of pediatric day surgery.

The secondary goal was to evaluate the influence of the anesthesiologist’s expertise in pediatrics on safety (in terms of rate of unplanned admissions) because there is, to our present knowledge, no data regarding this association in the literature.

## Methods

### Study design

Ethical approval for this study (ethics committee n° 1957_OPBG_2019) was provided by the Ethics Committee of Ospedale Pediatrico Bambino Gesù IRCCS, Rome, Italy (Chairperson Prof. G. Andria), on 18 September 2019. The requirement for written informed consent was waived by the Ethics Committee of Ospedale Pediatrico Bambino Gesù IRCCS. All methods were performed in accordance with the ethical standards as laid down in the Declaration of Helsinki and its later amendments or comparable ethical standards.

This is a retrospective analysis of a single institution’s experience, and patients’ confidentiality was protected.

All children who underwent day-case surgery procedures in anesthesia between 12 September 2012 and 18 April 2018 were included in the study. Inclusion criteria for day-case surgery were as follows: ASA physical status classification system grades I or II, length of surgery lower than 90, and procedures which do not require urinary catheter at the end of surgery. Exclusion criteria were as follows: newborns, obesity, ASA physical status classification system grade III or upper, potential difficult airway management, familiarity for sudden infant death syndrome, QT syndrome, asthma episode in the last month, procedures which cause bleeding up than 10% of blood volume, sleep apneas, and procedure with relevant pain at the end.

This manuscript adheres to the applicable Strengthening the Reporting of Observational Studies in Epidemiology (STROBE) guidelines (www.strobe-statement.org).

We made a retrospective analysis of the hospital database and focused on children requiring unplanned admission to the hospital for the night. Admission in hospital we implied as any admission from day-case surgery unit to the historical headquarters. Investigators identified unplanned admissions by the software packages “OBG Clinico®” and “GSED®,” which provides all database about patients of Ospedale Pediatrico Bambino Gesù. We also investigated distribution of patients about kind of surgery and experience in pediatrics of anesthesiologists. The experience of anesthesiologists was divided in three classes: less than 5 years of experience in pediatrics, between 5 and 15 years of experience in pediatrics, and more than 15 years of experience in pediatrics.

Anesthesiologists and surgeons selected patients the day before surgery investigating clinical history and objective examination (excluding patients with respiratory infections) and gave informational materials in addition to the information material that the families received at the time of the first surgical visit which indicated the procedure.

To evaluate families and patient’s education, anesthesiologists used a checklist (Table 1) to assess 10 parameters. During the preoperative examination on the day before the procedure, the anesthesiologist used the checklist at the end of the visit to complete the training of parents or caregivers and test their level of understanding. For example, it was explained to parents or caregivers that before discharge from the day surgery unit, the risk of patients falling was high, and the instructions of the health personnel had to be followed.

**Table 1:** Families and patients learning checklist

	**Learning barrier**	**Subject of learning**	**Methods**	**Level of learning**	**Comments**	**Operator signature**
Knowledge of disease						
Drugs use (indication and collateral effects)						
Drugs use (way of administration and dilution)						
Pain control						
Risk of falling						
Food-drugs interaction						
Feed						
Infection prevention						
Washing of the hands						
Personal hygiene						

Families and patients learning checklist. One “inadequate” item is enough to cancel day-case surgery

Learning barrier: 0 no one, 1a deafness, 1b blindness, 1c cognitive deficit, 2a confusion, 2b disorientation, 3a anxiety, 3b depression, 3c agitation, 4 language, 5 instruction, 6 will

Subject of learning: *P* patient, *F* family, *A* other

Methods: *V* oral, *S* written, *D* practical demonstration, *A* other

Level of learning: *A* adequate, *I* inadequate

If one or more parameters resulted “inadequate,” the procedure was performed in the main venue of the hospital. The anesthesiologist was responsible for assessing the suitability of parents or caregivers’ level of education, understanding, and compliance with rules, through the use of the checklist (Table 1).

Parents or caregivers signed the informed anesthetic and surgical consent at the end of the visit. After the procedure and at the discharge from the surgical unit, a prescription for analgesia was filled. Potential postoperative complications were explained to the family, and the phone number of an advice and support line was provided. For this reason, the first night after surgery, patients and their families must stay overnight in the proximity of the hospital (100 km) providing, for example, to stay in a hotel for those patients who live far away. The parents, or legal guardians of the patients, are informed about the need to stay overnight for the first postoperative night within 100 km of the hospital and undertake to respect this rule.

The day after the surgery, patients returned to the “San Paolo” venue for a surgical control and medications to evaluate pain and minor surgical complications. The surgeon has the responsibility to discharge the patient after his evaluation and to contact any missing patients.

### Population

All children who underwent day-case surgery procedures under anesthesia between 12 September 2012 and 18 April 2018 were included in the study.

Anesthesia technique consisted in a general anesthesia with sevoflurane and O_2_/N_2_O (without neuromuscular blocking drugs), associated with topic and local anesthesia, or peripheric nerve blocks by ropivacaine 0.2% (with maximum dose 2 mg/kg), for avoiding opioids. For postoperative analgesia, paracetamol iv or paracetamol/tramadol was administered. Standard intraoperative monitoring required the use of heart rate (HR), oxygen peripheral saturation (SpO_2_), electrocardiogram (EKG), noninvasive blood pressure, temperature, and CO_2_ capnography and then continued in recovery rooms.

All patients were in spontaneous breathing with facial mask or laryngeal mask, and, in case of dentistry, oral intubation was performed.

Discharge criteria from the recovery room were as follows: Aldrete score 9 or equal to the preoperative evaluation. The discharge criteria from the day-case surgery unit were as follows: Aldrete score 10 or equal to the preoperative evaluation, hemodynamic stability, the presence of protecting reflexes, good mobilization, pain score less than 4 of face legs, activity, cry, consolability (FLACC) scale or visual analogic scale (VAS), no PONV, and no requirement of urinary catheter.

### Measures

The unplanned admission rate, the anesthetic reasons, and the correlation with the experience of the anesthesiologist were evaluated.

Anesthetic reasons for ward admission after surgery were perioperative complications: bronchospasm, laryngospasm, hemodynamic instability, anaphylaxis, PONV, and pain (with a FLACC or VAS score upper than 4). We implied perioperative complications as events that required immediate intervention, as they were potentially dangerous for the patient, and that after the procedure provoked an Aldrete score < 10 or less than the preoperative evaluation, or which did not allow the discharge criteria from the day-case surgical unit.

### Outcomes

The primary endpoint was the rate of unplanned admission following day-case surgery, to evaluate the system level of patient safety. The secondary goal was to evaluate the influence of the anesthesiologist’s expertise in pediatrics on safety (in terms of rate of unplanned admissions).

### Statistical analysis

Categorical data are presented as number and percentage and continuous data as median range. The chi-square test for association was used to determine if there is any association between procedures performed over the years and the different levels of experience in pediatrics. The null hypothesis is that the study variables are not associated (i.e., independent).

## Results

**Fig. 1:**
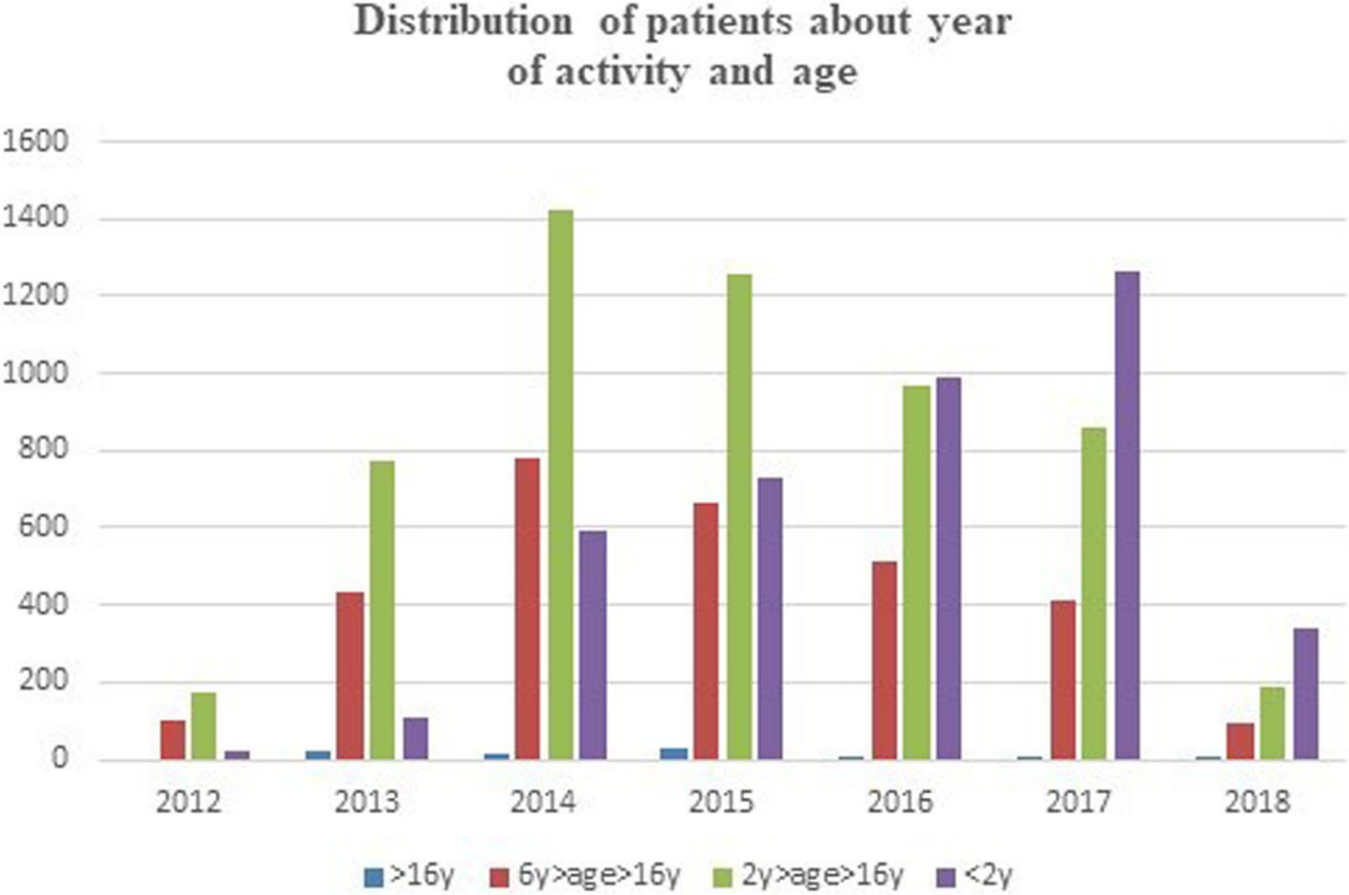
Showing the distribution of patients about year of activity and age expressed in years. From 2012, patients under 2 years of age are constantly increasing

From 12 September 2012 to 18 April 2018, 8826 day-case surgery procedures were performed under general anesthesia. The median age of patients was 5.5 years (Table 2, Fig. 1). Kinds of specialties were as follows: urology 33.6%, plastic surgery 30.8%, general surgery 17.4%, dermatology 11.6%, dentistry 3.1%, orthopedics 1.6%, and digestive endoscopy 1.6% (Table 3).

**Table 2:** Distribution of patients about year of activity and age expressed in years

Age	2012	2013	2014	2015	2016	2017	2018
> 16 years	0	24	13	26	2	5	1
6 years > age < 16 years	104	431	781	664	511	412	97
2 years > age < 16 years	176	774	1426	1254	971	857	191
< 2 years	21	110	595	727	992	1264	340

This table shows the distribution of patients about year of activity and age expressed in years. It is important to note that the activity of 2018 is about only 4 months

**Table 3:** Distribution of day-case procedures by specialty

Specialty	2012	2013	2014	2015	2016	2017	2018	Total
Urology	2	87	576	752	679	719	151	2966
Plastic surgery	69	392	607	587	520	452	97	2724
General surgery	4	31	369	262	362	391	120	1539
Dermatology	91	234	160	134	138	199	73	1029
Orthopedics	0	24	53	30	17	15	6	145
Dentistry	0	0	0	0	46	187	46	279
Digestive endoscopy	0	0	44	17	38	38	7	144
Total	166	768	1809	1782	1800	2001	500	8826

Our study focused only on cases of unplanned admission related to anesthetic causes, leaving out those related to surgical causes (such as bleeding).

There was no occurrence of respiratory, cardiac, allergic, or neurological complications requiring unplanned admission. Only two patients needed hospitalization after surgery. The first one, male, 15 years old, operated of circumcision had a syncope (really a temporary hypotension with systolic arterial blood pressure less of 70 mmHg) in the recovery room, without neurological damage, which did not require drugs. For this patient was performed a general anesthesia combined with ultrasound-guided penile block, providing adequate postoperative analgesia (VAS score always less than 4) with intravenous paracetamol. The second patient, male, 11 years old, was operated of nevus excision and had a vomiting episode refractory to drugs (ondansetron 0.1 mg/kg iv) and fluid challenge. It should be noted that this patient received an opioid-free anesthesia, ensuring intraoperative analgesia with local anesthesia and in the postoperative period with intravenous paracetamol. In addition, oral fluid administration was only on demand until the vomiting episode.

Both patients were hospitalized for clinical observation and the day after were discharged home in good conditions.

The physicians involved in these two cases were pediatric anesthesiologists with more than 15 years of experience in pediatrics.

There were no cases of unplanned admissions of patients discharged from the day-case surgery unit and returned home, not even after the postoperative surgical control. Furthermore, there were no cases of missing patients at the surgical control the day after surgery.

**Fig. 2:**
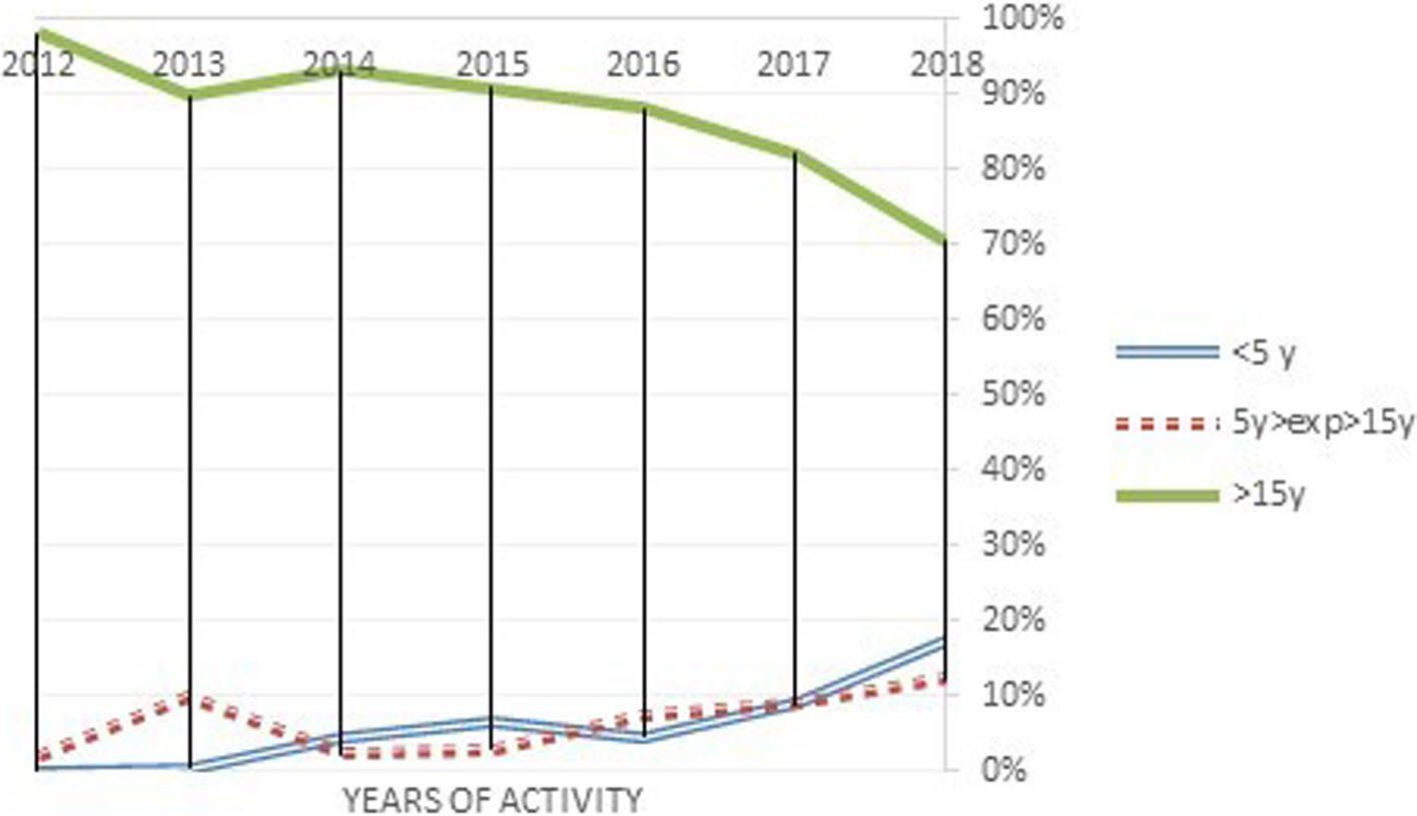
Trend of anesthesiologists’ activity by expertise

From the beginning, rate of procedures performed by anesthesiologists with less than 5 years of experience in pediatrics increased from 6.00% in 2012 to 22% in 2018 (Fig. 2). We observed the same trend for anesthesiologists with less than 15 years and more than 5 years of experience: from 1.8% (2012) to 8.4% (2018) (Fig. 2).

On the contrary, trend about physicians with more than 15 years of experience showed a decrease from 92.1% (2012) to 69.6% (2018) (Fig. 2). In other words, there has been a progressive reduction in the percentage of procedures performed by the most expert anesthesiologists over the years.

Results of chi-square tests were significant at the 5% level (*χ*^2^ = 363.982, *p* < 0.001). From this result, we may infer that there is a significant association between the service experience and the procedures performed in the years under study. The distribution of the number of procedures performed over the years is statistically significantly associated with the different levels of service experience (i.e., expertise).

## Discussion

Our retrospective analysis showed that Ospedale Pediatrico Bambino Gesù IRCCS has an extremely low rate of unplanned admissions related to anesthetic reasons. This data is crucial to reconcile an increase in the volume of day-case surgery procedures and maintaining a high standard of safety.

At Ospedale Pediatrico Bambino Gesù IRCCS, the decision to find a new facility dedicated to day-case surgery was inspired by the requirement to reduce costs and increase spaces for high complexity procedures in the historical headquarters. Furthermore, day-case procedures can reduce family and patient’s stress (Caredda et al. 2019).

It was a challenging innovation, because reducing costs should not impact on quality of assistance and safety. At the beginning, anesthesiologists involved in the project were largely physicians with a long experience in pediatrics. This fact was important not only for skills but also for the right selection of patients too. In fact, according to Italian guidelines for pediatric day-case surgery, preoperative tests were not required, so a precise medical history was essential for safety (Luca et al. 2018). Only when a detailed medical history was not available, preoperative tests were performed (Luca et al. 2018).

According to the literature, the rate of unplanned admissions for surgical and anesthetic reasons following a pediatric day-case surgery varies from 1.8 to 2.2% (Blacoe et al. 2008; Awad et al. 2004). This audit demonstrated a very low rate of incidence (0.023%; *CI* 95%, 0.0 ≤ *p* ≤ 0.0005) referred to anesthetic reasons. Despite other studies included otorhinolaryngologic procedures and we perform this kind of surgery in another venue, the unplanned admission rate at Ospedale Pediatrico Bambino Gesù IRCCS is lower than unplanned admission rate of these studies net of cases referred to ENT surgery (Blacoe et al. 2008). Furthermore, in our study, there are dentistry and digestive endoscopic procedures, during which, as for otorhinolaryngologic procedures, airway represents the most important area of working for both anesthesiologist and surgeon. Therefore, a very strict selection of patients and procedures provides a high-quality standard, with standardized discharge criteria (Jakobsson 2019; Lerman 2019).

It is remarkable to note that the only two complications involved anesthesiologists with a long experience, and that the increase of activity by new anesthesiologists did not reduce the quality of assistance. Furthermore, while the activity of young anesthesiologists increased, higher-risk procedures such as dentistry and digestive endoscopy were also introduced, without any increase of complications. The two complications are not statistically relevant, and the fact that they occurred to more experienced colleagues is only stated for completeness. However, the number of procedures that less experienced colleagues have carried out over the years is statistically significant: the distribution of these procedures, compared to those performed by more experienced colleagues, is statistically significant. This shows how the system has held up, in terms of safety, despite the entry of new anesthesiologists, keeping efficient the system level of patient safety.

It may be explained by the hospital’s inclusion protocol of young physicians. The protocol provides procedures without expert supervision after a period of supervision and attendance of skills and procedural courses. Procedural courses for young physicians concerned the following: correct identification of the patient, handover between physicians, preventions of fall in hospital, emergency communications, and management of drugs. In addition, all staff in the operating room obtained pediatric advanced life support (PALS) certification, provided by the American Heart Association, with a refresh course every 2 years. The trained personnel was also involved in high fidelity simulations every month, inspired by crisis resource management principles (Shapiro et al. 2014a). Debriefings after simulations disclosed weakness about communication, guidelines application, logistic organization, and time of reaction. The results of debriefing were communicated to the chief anesthesiology to improve the safety and quality of assistance, working about staff education, write protocols, and optimize resources.

Secondly, young physicians always worked with an expert anesthesiologist in the operating theater, to balance experience, and age decline with inexperience and psychophysical prowess (Giacalone et al. 2016). According to the literature, the expertise of the anesthesiologist can improve the safety of anesthesia (Habre et al. 2017; Graaff et al. 2021). Conversely, other studies suggest that the age of anesthesiologists could reduce performance, vigilance, and fine motor movements (Giacalone et al. 2016). Moreover, the anesthesiologist is not only the relevant variable in pediatric patient’s safety but also the quality and volume of the facility are crucial too. Furthermore, the Ospedale Pediatrico Bambino Gesù IRCCS, being the largest pediatric hospital in Europe, is part of the training network for residents of several Italian universities. Therefore, the hospital anesthesiologists are always supported by residents who can perform anesthesia only under the direct supervision of the pediatric anesthesiologist. This datum further reinforces the importance of the efficiency of the hospital system with respect to the experience of the individual physician.

Another interesting point is that even if pain is the most important factor in unplanned admission, and pain is often undertreated in pediatrics (Marchetti et al. 2023), in our experience, no patients were admitted for pain (Awad et al. 2004; Shapiro et al. 2014b). Probably, the combination of locoregional anesthesia and mini-invasive surgical incisions reduced pain substantially (Lerman 2019). Furthermore, there were no unplanned admissions, not even of patients discharged from the day-case surgery unit, i.e., from home. This means that family education has been effective, as postoperative pain management is entirely the responsibility of the family. Therefore, in this data, the implementation of a family educational program and the use of a structured checklist have a strong impact (Shapiro et al. 2013). Indeed, the training of families and patients education provided basic knowledge on safety about fasting and control of complications at home and enhanced a good collaboration with medical and nurse staff (Hanna and Mason 2012).

Moreover, opioid-free anesthesia decreased the incidence of PONV (one single case), which is the second main anesthetic reason for unplanned admission according to literature (Shapiro et al. 2014b). In addition, vomiting in pediatrics after day-case surgery can be explained by a compulsive administration of liquids after surgery to stimulate urine output (Gan et al. 2014). Actually, a zero balance regimen was chosen for the study, and liquids were administered to children only on demand (clinical and monitored signs of hypoperfusion) to reduce this occurrence (Apfel et al. 2012).

Another factor of success could be the adoption of a very strict score for admission to surgery, selecting patients without pathologies capable of generating complications, with a clear medical history, and less risky procedures, thus excluding ENT surgery (Amoils et al. 2016; Mitchell et al. 2016). In the same way, temporal criteria were not used for discharge but only objectionable clinical criteria (Aldrete score, hemodynamic stability, presence of protecting reflexes, good mobilization, no pain, no requirement of urinary catheter) (Jakobsson 2019; Lerman 2019).

### Limitations

The present study has several limitations. The first is to be a retrospective study. However, many of the studies on the same or similar topic are retrospective studies. The quality of our data is ensured by the fact that the software of our hospital that store the data automatically have been queried. Obviously, programming a prospective study could guarantee a higher level of quality and allow for the real-time analysis of sentinel events. The second limitation relates to the case mix. In fact, ENT-type procedures are not considered in our study, as it happens in other studies. However, it should be noted that the venue dedicated to day-case surgery is totally detached from the main hospital venue, without some essential services. For this reason, ENT-day-case surgery is reserved for a venue where supplementary services, such as the intensive care unit, are provided. The third limitation of our study is that it is a single-center study. However, this limitation also has a high value, highlighting the strengths and weaknesses of an organizational model that could be replicated in other centers.

## Conclusions

Day-case surgery in pediatrics can be a good choice to improve saving and reduce stress in children, without affecting the quality of assistance. It can also be implemented in a dedicated venue, with a standardized protocol for unplanned admissions. Good quality of patient’s selection (performing low-risk procedures in low-risk patients with well-selected parents), the structural safety, family educational programs, and the hospital organizational model with trainings for anesthesiologists can improve global safety of anesthesia for day-case surgery. The experience of anesthesiologists can be a secondary factor on safety in hospitals with effective educational programs and an inclusion protocol of young physicians.

## Data Availability

The datasets used and/or analyzed during the current study are available from the corresponding author on reasonable request.

## Abbreviations

EKG: Electrocardiogram
ENT: Ear, nose and throat
FLACC: Face, legs, activity, cry, and consolability
HR: Heart rate
PALS: Pediatric Advanced Life Support
PONV: Postoperative nausea and vomiting
SpO_2_: Oxygen peripheral saturation
VAS: Visual analogic scale
